# Our Trip to Cincinnati

**Published:** 1880-04-20

**Authors:** 


### *OUR TRIP TO CINCINNATI.*

The managing Editor of this Journal had the good fortune to be among the invited guests in the late great excursion to the Queen city of the West. A trip of nearly 500 miles passing over two great roads, the Western & Atlantic to Chattanooga and the Cincinnati Southern to Cincinnati. The former of these roads, under the able supervision of Gen. McRae, is in splendid condition, the entire equipment and management evincing the work of a master mind. The great Cincinnati Southern, a new road just completed, is also in splendid condition, and is one of the most gigantic human enterprises ever accomplished.

It is scarcely appropriate in a medical journal—nor does our space permit—to detail the many interesting scenes and incidents of the great excursion; the deep cuts, the high embankments and lofty bridges; the twenty-five tunnels, the cliffs, precipices, gorges and cataracts; the magnificent mountain scenery, charming vallies, varied and beautiful landscapes; and then, on arrival, the wonderful display of fire-works, the grand reception, the generous hospitality, the sumptuous banquet, the soul-stirring music and the varied attractions of Cincinnati; these have all been made familiar to the public through the newspapers of the country.

During our stay in the City it was our privilege to enjoy special courtesies from a number of the citizens. Among these we are pleased to mention our brother of the quill, Dr. J. C. Culbertson, one of the editors of that excellent journal the *Lancet & Clinic*. Through his kindness we were given a delightful and exhilarating drive, visiting many attractive scenes and resorts, among which were the Zoological Garden with its varied and interesting display of animals, birds, etc., and the adjacent suburban parks and groves with their green sward, beautiful walks and magnificent residences, giving evidence of taste, refinement and luxurious wealth.

We had the pleasure also of a social interview with Dr. John A. Murphy, the distinguished Professor of Practice in the Miami Medical College, who extended us many courtesies, and showed us Cincinnati's great Hospital, its structure, its wards and its admirable management.

We also saw Prof. Bramble, the Dean of the Cincinnati College of Medicine and Surgery, who, though confined to his room, with sickness, received us with marked cordiality. We had likewise a social interview with Dr. W. W. Seeley, Prof. of Ophthalmology in the Medical College of Ohio, through whose kindness we were shown the building of that Institution, its museum, laboratory, etc.

*The Drug Houses of Cincinnati.*—We had time only to examine a few of these, and found that a large business in this line is done in Cincinnati. We are indebted to Prof. John M. Lloyd, of the pharmaceutical establishment of Merrill, Thorpe & Lloyd, for special courtesies. He politely and kindly showed us his working laboratory where his fluid extracts and tinctures are prepared, disclosing extensive machinery, percolators and apparatus for the preparation of medicines, to which he gives constant and careful attention. He visited with us Cincinnati's great Library and other places of interest. He is a man of intelligence and energy, and the members of the firm with which he is connected are active, polite and efficient business men.

See their advertisement in this Journal.

*Surgical Instruments, Batteries, etc.*—We met with a very pleasant reception from Mr. Autenrieth, manufacturer of *Surgical and Orthopedical Instruments*, abdominal supporters, trusses, etc. A fine establishment and an excellent business man. See his advertisement.

Among the importers and wholesale druggists and manufacturing chemists of Cincinnati, we mention with pleasure the *House of W. S. Merrill & Co.* It is a large and well conducted establishment, and the gentlemen connected with it polite, active and accommodating business men. An advertisement from this House may be found in the present issue of the Journal.

LOUISVILLE.—On our return we stopped at the beautiful and attractive city of Louisville. Our stay there was brief, but we had the good fortune to meet and enjoy a pleasant interview with Prof. L. P. Yandell, through whose kindness we were permitted to examine University College building, museum, etc. We visited also the Kentucky School of Medicine, and found the spring session progressing, and enjoyed two interesting lectures; one by Prof. Coomes, on [illegible]hysiology; the other by Prof. Mathews, on Surgical Pathology.

We are indebted to Mr. Wyly Wilson, an intelligent student in this school, for an introduction and an agreeable interview with Prof. Mathews, and for other courtesies.

